# Supplementary material for: Plastome phylogenomic study of Gentianeae (Gentianaceae): widespread gene tree discordance and its association with evolutionary rate heterogeneity of plastid genes
Source: BMC Plant Biol. 2020 Jul 17;20:340. doi: 10.1186/s12870-020-02518-w (PMC7368685; doi:10.1186/s12870-020-02518-w)
Supplement: Supplementary file 1 — Additional file 1: Figure S1. Loss of rpl33 coding region in plastomes of Comastoma pulmonarium and Swertia hispidicalyx. There is a stop codon in coding region of rpl33 in Comastoma pulmonarium due to the change of cytosine (C) to thymine (T) at 22 bp, and a small deletion containing the coding region of rpl33 between the psaJ and rps18 gene in the plastome of Swertia hispidicalyx. Figure S2. Maximum likelihood phylogram of Gentianeae from unpartitioned concatenated matrix of 76 plastid protein-encoding genes using RAxML. Maximum likelihood bootstrap (BS) values and the PP calculated from MrBayes are shown at nodes, except nodes with 100% BS and 1.0 PP. Figure S3. Phylogenetic informativeness profile estimated in PhyDesign. (A) The ultramteric tree of Gentianeae. (B) Net phylogenetic informativeness profile for 76 plastid protein-coding genes. Ten genes with the greatest informativeness are color-coded and indicated at the right. X- and Y-axes represent relative-time and net phylogenetic informativeness, respectively. Table S1. Taxa included in present study. Table S2. Genetic characteristics of 76 protein-coding genes. [file 12870_2020_2518_MOESM1_ESM.pdf]

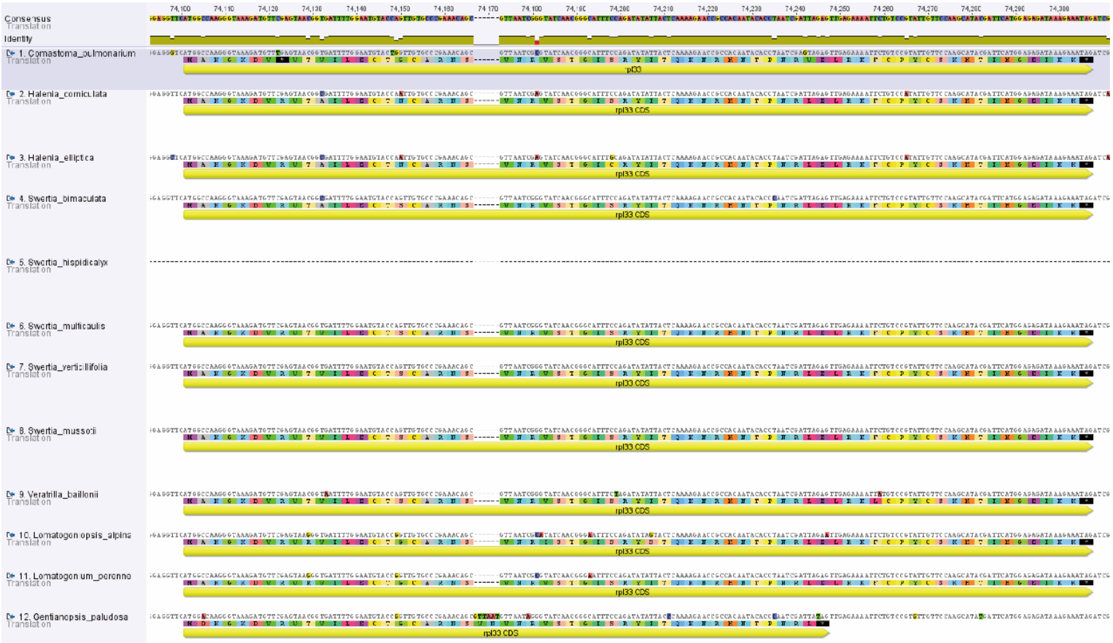

Figure S1 Loss of *rpl33* coding region in plastomes of *Comastoma pulmonarium and Swertia hispidicalyx*. There is a stop codon in coding region of *rpl33* in *Comastoma pulmonarium* due to the change of cytosine (C) to thymine (T) at 22bp, and a small deletion containing the coding region of rpl33 between the *psaJ* and *rps18* gene in the plastome of *Swertia hispidicalyx*.

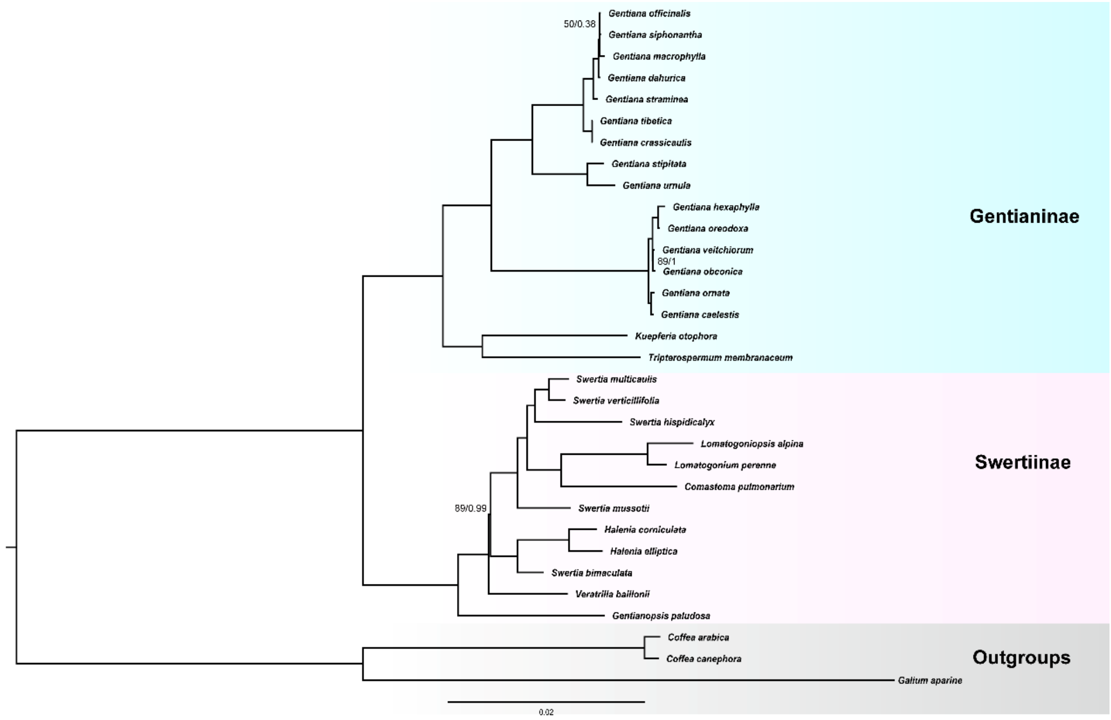

Figure S2 Maximum likelihood phylogram of Gentianeae from unpartitioned concatenated matrix of 76 plastid protein-encoding genes using RAxML. Maximum likelihood bootstrap (BS) values and the PP calculated from MrBayes are shown at nodes, except nodes with 100% BS and 1.0 PP.

A

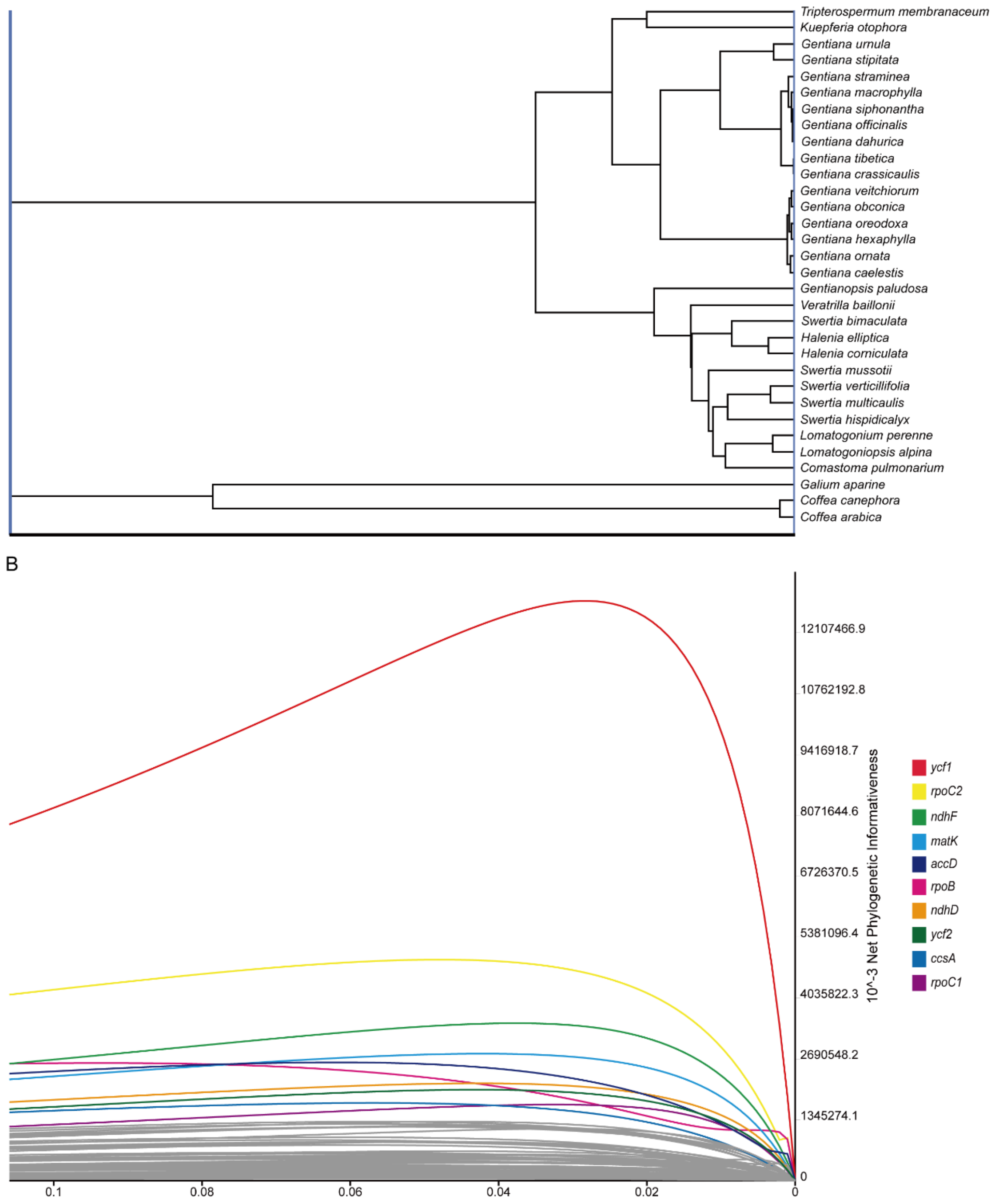

Figure S3 Phylogenetic informativeness profile estimated in PhyDesign. (A) The ultramteric tree of Gentianeae. (B) Net phylogenetic informativeness profile for 76 plastid protein-coding genes. Ten genes with the greatest informativeness are color-coded and indicated at the right. X- and Y-axes represent relative-time and net phylogenetic informativeness, respectively.

Table S1 Taxa included in present study. NCBI accession numbers and voucher specimens' information are provided for newly sequenced plastomes, "-" indicates no applicable

**Family**	**Subtribes**	**Species**	**NCBI accession numbers**	**Voucher specimens information**
Gentianaceae	Swertiinae	*Comastoma pulmonarium*	MT228723	FSC-342
Gentianaceae	Swertiinae	*Gentiana urnula*	MT228724	ZJW6386
Gentianaceae	Swertiinae	*Gentianopsis paludosa*	MT228725	FSC-55
Gentianaceae	Swertiinae	*Halenia elliptica*	MT228726	FSC-339
Gentianaceae	Swertiinae	*Lomatogoniopsis alpina*	MT228728	ZJW5972
Gentianaceae	Swertiinae	*Lomatogonium perenne*	MT228729	ZJW5118
Gentianaceae	Swertiinae	*Swertia multicaulis*	MT228730	ZJW5106
Gentianaceae	Swertiinae	*Veratrilla baillonii*	MT228732	ZJW6591
Gentianaceae	Swertiinae	*Halenia corniculata*	MK606372.1	-
Gentianaceae	Swertiinae	*Swertia hispidicalyx*	MH321887.1	-
Gentianaceae	Swertiinae	*Swertia mussotii*	KU641021.1	-
Gentianaceae	Swertiinae	*Swertia verticillifolia*	MF795137	-
Gentianaceae	Swertiinae	*Swertia bimaculata*	MH394374	-
Gentianaceae	Gentianinae	*Kuepferia otophora*	MT228727	FSC-76
Gentianaceae	Gentianinae	*Tripterospermum membranaceum*	MT228731	KUN1220748
Gentianaceae	Gentianinae	*Gentiana caelestis*	MG192304.1	-
Gentianaceae	Gentianinae	*Gentiana crassicaulis*	KJ676538.1	-
Gentianaceae	Gentianinae	*Gentiana dahurica*	MH261259.1	-
Gentianaceae	Gentianinae	*Gentiana hexaphylla*	MG192305.1	-
Gentianaceae	Gentianinae	*Gentiana macrophylla*	KY856959.1	-
Gentianaceae	Gentianinae	*Gentiana obconica*	MG192306.1	-
Gentianaceae	Gentianinae	*Gentiana officinalis*	MH261261.1	-
Gentianaceae	Gentianinae	*Gentiana oreodoxa*	MG192307.1	-
Gentianaceae	Gentianinae	*Gentiana ornata*	MG192308.1	-
Gentianaceae	Gentianinae	*Gentiana siphonantha*	MH261260.1	-
Gentianaceae	Gentianinae	*Gentiana stipitata*	MG192309.1	-
Gentianaceae	Gentianinae	*Gentiana straminea*	KJ657732.1	-
Gentianaceae	Gentianinae	*Gentiana tibetica*	KU975374.1	-
Gentianaceae	Gentianinae	*Gentiana veitchiorum*	MG192310.1	-
Rubiaceae	-	*Coffea arabica*	NC_008535.1	-
Rubiaceae	-	*Coffea canephora*	KU500324.1	-
Rubiaceae	-	*Galium aparine*	KY562587.1	-

Table S2 Genetic characteristics of 76 protein-coding genes used in analyses, including nonsynonymous rate (d*N*), Synonymous rate (d*S*), nucleotide diversity (π), percent variability (PV), phylogenetic informativeness (PI), gene-tree discordance (GD) and partitioned coalescence support (PCS). Detailed information of functional group is provided in **Table 2**

Genes	Functional groups	d*N*	d*S*	d*N*/d*S*	π	PV	PI	GD	PCS
accD	OG	0.3653	1.0710	0.3411	0.0590	0.2228	67.5645	2.1426	52
atpA	ATP	0.0551	1.1528	0.0478	0.0343	0.1808	36.1631	8.7252	-56
atpB	ATP	0.0514	0.7176	0.0716	0.0270	0.1514	21.5446	2.0286	-56
atpE	ATP	0.1797	0.7273	0.2471	0.0392	0.2114	8.7099	17.6664	2
atpF	ATP	0.2248	0.7315	0.3073	0.0438	0.2250	15.2736	14.5917	27
atpH	ATP	0.0056	1.1477	0.0049	0.0291	0.1564	4.5894	32.8122	52
atpI	ATP	0.0924	0.7713	0.1197	0.0331	0.1748	13.1140	0.9808	48
ccsA	OG	0.4041	1.1645	0.3470	0.0658	0.2811	55.2155	0.8087	56
cemA	OG	0.3444	0.7356	0.4682	0.0517	0.2727	23.1032	13.3351	27
clpP	OG	0.1693	0.6523	0.2596	0.0436	0.1881	17.6351	15.7227	0
matK	OG	0.5333	1.2032	0.4433	0.0824	0.3216	87.6416	0.9279	4
ndhA	NDH	0.232	0.9876	0.2349	0.0524	0.2423	38.8263	5.5621	0
ndhB	NDH	0.0412	0.1099	0.3748	0.0129	0.0301	6.7568	13.6284	0
ndhC	NDH	0.1199	0.9808	0.1223	0.0361	0.1889	7.9042	22.433	0
ndhD	NDH	0.1904	1.0327	0.1844	0.0694	0.0537	62.1701	2.6858	0
ndhE	NDH	0.1535	1.0038	0.1530	0.0448	0.1799	9.7540	17.3181	0
ndhF	NDH	0.2168	1.1928	0.1818	0.0603	0.0271	109.9486	3.9719	0
ndhG	NDH	0.2711	1.0581	0.2562	0.0551	0.2689	19.9161	10.7515	0
ndhH	NDH	0.1388	1.1791	0.1177	0.0490	0.2152	36.1738	4.5341	0
ndhI	NDH	0.1511	1.2747	0.1185	0.0501	0.2032	17.8783	5.1046	0
ndhJ	NDH	0.1261	0.8440	0.1494	0.0410	0.1983	11.5506	9.5790	0
ndhK	NDH	0.1310	0.9647	0.1358	0.0376	0.1856	15.4820	4.9396	0
petA	PET	0.1236	0.9438	0.1309	0.0436	0.2090	21.9469	3.5395	-56
petB	PET	0.0344	1.0109	0.0340	0.0279	0.1376	14.6061	8.4695	2
petD	PET	0.0554	0.8376	0.0662	0.0321	0.1303	10.2926	23.1140	-52
petG	PET	0.0270	0.5722	0.0473	0.0207	0.1351	1.1984	31.7335	8
petL	PET	0.1336	0.7772	0.1719	0.0303	0.2151	1.3856	35.6260	0
petN	PET	0.0961	0.5843	0.1645	0.0293	0.1379	1.5554	29.4858	-24
psaA	PSA	0.0326	0.7722	0.0422	0.0249	0.1409	35.2175	6.7164	0
psaB	PSA	0.0292	0.8319	0.0351	0.0268	0.1367	34.2358	3.2928	-56
psaC	PSA	0.0273	1.1449	0.0238	0.0257	0.1276	5.2226	17.3406	54
psaI	PSA	0.1990	0.3948	0.5040	0.0266	0.1759	1.8664	16.8629	-27
psaJ	PSA	0.0566	0.7561	0.0748	0.0326	0.1778	4.3983	28.5362	30
psbA	PSB	0.0151	0.8314	0.0182	0.0252	0.1246	16.5847	15.3867	28
psbB	PSB	0.0569	0.8398	0.0678	0.0278	0.1594	25.213	7.2645	1
psbC	PSB	0.0281	0.7164	0.0393	0.0260	0.1438	21.5323	9.8471	28
psbD	PSB	0.0205	0.6772	0.0303	0.0223	0.118	14.3902	10.2727	-56

psbE	PSB	0.0376	0.5819	0.0646	0.0244	0.1245	2.8053	17.5954	-25
psbF	PSB	0.0484	0.6442	0.0751	0.0229	0.1453	1.5568	34.5233	-2
psbH	PSB	0.2929	0.6900	0.4245	0.0471	0.2237	8.1256	17.1520	56
psbI	PSB	0.0388	0.6232	0.0622	0.0288	0.1389	1.4368	26.5306	54
psbJ	PSB	0.0565	0.4395	0.1286	0.0165	0.1000	1.2624	32.8013	53
psbL	PSB	0.0509	0.3419	0.1487	0.0156	0.0877	1.1146	34.6862	-27
psbM	PSB	0.0917	0.5728	0.1601	0.0295	0.1471	1.3478	28.6185	26
psbN	PSB	0.0565	0.4743	0.1191	0.0192	0.1163	1.4453	28.3032	2
psbT	PSB	0.0001	0.9968	0.0001	0.0336	0.1143	2.4292	29.2809	48
psbZ	PSB	0.0737	0.8185	0.0900	0.0223	0.1882	2.4807	29.7000	0
rbcL	Rubisco	0.0816	0.8366	0.0976	0.0294	0.1505	37.4035	2.2135	56
rpl14	RPL	0.0878	0.8567	0.1025	0.0380	0.1995	5.4226	9.7891	-52
rpl16	RPL	0.1220	1.2469	0.0978	0.0390	0.2034	9.1429	16.4541	55
rpl2	RPL	0.0295	0.1926	0.1529	0.0098	0.0624	3.0633	25.9169	50
rpl20	RPL	0.2510	0.9705	0.2586	0.0449	0.2164	16.7373	15.1967	0
rpl22	RPL	0.3859	1.7402	0.2218	0.0683	0.2952	25.7191	2.4413	4
rpl23	RPL	0.0236	0.2666	0.0885	0.0079	0.0609	0.9822	32.11	-26
rpl32	RPL	0.2438	1.4596	0.1670	0.0535	0.2222	7.6006	21.9948	28
rpl33	RPL	0.4289	1.3109	0.3249	0.0498	0.1324	9.2624	NA	-24
rpl36	RPL	0.0609	1.2482	0.0488	0.0335	0.2342	1.7339	31.2538	54
rpoA	RPO	0.2720	1.0983	0.2476	0.0547	0.2653	34.6309	16.6631	-56
rpoB	RPO	0.1229	0.8775	0.1401	0.0357	0.2036	69.2034	3.2748	-28
rpoC1	RPO	0.1382	0.9017	0.1533	0.0381	0.3872	46.1063	8.1058	-56
rpoC2	RPO	0.2508	0.9633	0.2603	0.0526	0.2509	147.8433	0	56
rps11	RPS	0.1891	1.154	0.1638	0.0498	0.2160	13.9206	17.0221	56
rps12	RPS	0.0423	0.3696	0.1144	0.0187	0.0780	4.0176	27.9498	-56
rps14	RPS	0.158	0.8777	0.1800	0.0435	0.2400	5.9692	12.1429	0
rps15	RPS	0.2841	1.4808	0.1919	0.0627	0.2500	11.7144	5.4910	0
rps18	RPS	0.1622	0.7838	0.2070	0.0386	0.1883	6.5995	19.0925	-28
rps19	RPS	0.1347	0.9256	0.1456	0.0408	0.2174	4.8625	21.3507	56
rps2	RPS	0.1390	0.9439	0.1472	0.0365	0.2076	15.7626	10.3947	0
rps3	RPS	0.2297	1.2682	0.1811	0.0548	0.2861	19.7711	7.1380	27
rps4	RPS	0.1255	0.8958	0.1401	0.0394	0.2085	13.7031	6.4906	0
rps7	RPS	0.0196	0.1704	0.1152	0.0077	0.0473	1.2825	20.4554	50
rps8	RPS	0.2600	1.0960	0.2372	0.0568	0.2990	13.6557	15.5247	-24
ycf1	OG	0.7634	1.2515	0.6100	0.0884	0.3460	438.8369	0.9808	-56
ycf2	OG	0.1163	0.1621	0.7173	0.0191	0.0974	58.8030	1.0214	-28
ycf3	OG	0.0429	0.6762	0.0635	0.0243	0.1310	6.1739	14.5601	4
ycf4	OG	0.2270	0.7662	0.2963	0.0393	0.2283	14.4875	7.2522	-52
